# Hunting for Bileptons at Hadron Colliders

**DOI:** 10.3390/e26100850

**Published:** 2024-10-08

**Authors:** Gennaro Corcella

**Affiliations:** INFN, Laboratori Nazionali di Frascati, Via E. Fermi 54, 00044 Frascati, Italy; gennaro.corcella@lnf.infn.it

**Keywords:** BSM phenomenology, bileptons, hadron colliders

## Abstract

I review possible signals at hadron colliders of bileptons, namely doubly charged vectors or scalars with lepton number L=±2, as predicted by a 331 model, based on a $SU{\left(3\right)}_{c}\times SU{\left(3\right)}_{L}\times U{\left(1\right)}_{X}$ symmetry. In particular, I account for a version of the 331 model wherein the embedding of the hypercharge is obtained with the addition of three exotic quarks and vector bileptons. Furthermore, a sextet of $SU{\left(3\right)}_{L}$, necessary to provide masses to leptons, yields an extra scalar sector, including a doubly charged Higgs, i.e., scalar bileptons. As bileptons are mostly produced in pairs at hadron colliders, their main signal is provided by two same-sign lepton pairs at high invariant mass. Nevertheless, they can also decay according to non-leptonic modes, such as a TeV-scale heavy quark, charged 4/3 or 5/3, plus a Standard Model quark. I explore both leptonic and non-leptonic decays and the sensitivity to the processes of the present and future hadron colliders.

## 1. Introduction

The Standard Model (SM) of electroweak and strong interactions is a complete theory, but it exhibits several drawbacks, such as the hierarchy problem in the Higgs sector, neutrino masses, or Dark Matter, which call for a theory with a more general gauge structure and possibly new particles. As well-motivated SM extensions, such as supersymmetry or extra dimensions, have provided no visible signal at the LHC thus far, it is mandatory to explore alternative scenarios. In this paper, I review the work carried out in the last few years [1,2,3] in the framework of the $SU{\left(3\right)}_{L}\times SU{\left(3\right)}_{C}\times U{\left(1\right)}_{X}$ model [4,5,6], also known as the 331 model, and its possible signals at the Large Hadron Collider (LHC) and at a future 100 TeV hadron collider (FCC-hh).

Among its main features, this model predicts the existence of bileptons, i.e., gauge bosons $\left(Y^{--},Y^{++}\right)$ of charge Q=±2 and lepton number L=±2, which is why one often refers to it as a bilepton model. Furthermore, in the specific formulation of [4], one is capable of explaining the asymmetry of the third quark family, i.e., top and bottom quarks, with respect to the other two, while the existence of three families, i.e., $N_{f}=N_{C}=3$, $N_{C}$ being the number of colours, is a consequence of the requirement of an anomaly-free theory (see also the detailed discussion in [7]).

As will be detailed later on, the scenario that will be investigated, besides the vectors $Y^{++(--)}$, predicts a number of new particles, which may possibly be within reach of the LHC or a future hadron collider, such as FCC-hh. Among those, one has heavy quarks with charge 5/3, usually labelled *T*, or charge 4/3, i.e., *D* or *S*, which typically have a mass of the order of a few TeVs (see the analysis in [3]). Moreover, a complete description of the model requires the inclusion of a Higgs sector, which is a sextet of $SU{\left(3\right)}_{L}$ and is needed to provide mass to the leptons. In the Higgs sector, the prediction of new doubly charged scalars is particularly relevant. Such Higgs-like bosons with charge ±2 have been intensively searched by the experimental collaborations in different new physics models, setting mass bounds between 900 and 1100 GeV [8,9] at $\sqrt{s}=13$ TeV and integrated luminosities L=139 fb^−1^ and 12.9 fb^−1^, respectively. As far as I know, no specific search for vector bileptons has been undertaken so far.

On the other hand, as will be detailed in the following, Refs. [1,3] investigated the phenomenology of vector bileptons, decaying into leptonic or non-leptonic final states, while Ref. [2] explored both vector and scalar bileptons, concentrating on final states with same-sign lepton pairs. All such papers published results for reference points that are not yet excluded by the experimental searches, with a bilepton mass just below the exclusion range, in order to maximize the production cross section.

The plan of this contribution is the following. In Section 2, I shall review the main ingredients of the 331 model, in the version proposed in [4]. In Section 3, I shall critically present the phenomenological results contained in Refs. [1,2,3]. In Section 4, some concluding remarks will be presented.

## 2. Theoretical Framework

Following Ref. [4], the gauge structure of the bilepton model is $SU{\left(3\right)}_{c}\times SU{\left(3\right)}_{L}\times U{\left(1\right)}_{X}$, with the fermions (quarks) in the fundamental of $SU{\left(3\right)}_{c}$ arranged into triplets of $SU{\left(3\right)}_{L}$. As anticipated in the Introduction, the third quark family (top and bottom) is treated asymmetrically with respect to the first two families in the electroweak $SU{\left(3\right)}_{L}$. In detail, as for the first two families, one has

(1) $$Q_{1}=\left(\begin{array}{c} u_{L}\\ d_{L}\\ D_{L} \end{array}\right),\;Q_{2}=\left(\begin{array}{c} c_{L}\\ s_{L}\\ S_{L} \end{array}\right),\;Q_{1,2}\in \left(3,3,-1/3\right)$$

under $SU{\left(3\right)}_{c}\times SU{\left(3\right)}_{L}\times U{\left(1\right)}_{X}$, while, for the third one, it is

(2) $$Q_{3}=\left(\begin{array}{c} b_{L}\\ t_{L}\\ T_{L} \end{array}\right),\;Q_{3}\in \left(3,\bar{3},2/3\right).$$

In the above formulation, *D*, *S*, and *T* are quarks, with charge 4/3 (*D* and *S*) or 5/3 (*T*). In the following, I will explore scenarios wherein such quarks are either within or outside the reach of present and future hadron colliders.

The right-handed quarks ($\bar{q}$), as happens in the SM, are singlets even under $SU{\left(3\right)}_{L}$. Their representations are the following:

(3) $$\begin{array}{cc} \left(d_{R},s_{R},b_{R}\right) & \in (\bar{3},1,1/3) \end{array}$$

(4) $$\begin{array}{cc} \left(u_{R},c_{R},t_{R}\right) & \in (\bar{3},1,-2/3) \end{array}$$

(5) $$\begin{array}{cc} \left(D_{R},S_{R}\right) & \in (\bar{3},1,4/3) \end{array}$$

(6) $$\begin{array}{cc} T_{R} & \in (\bar{3},1,-5/3). \end{array}$$

One can notice that adding such new particles to the Standard Model states is not enough to cancel the $SU{\left(3\right)}_{L}$ anomalies [4,7]. Therefore, one has to introduce new leptonic states in three $\bar{3}$ representation. As a result, the three lepton families, unlike the quarks, are arranged in a ‘democratic’ manner as triplets of $SU{\left(3\right)}_{L}$:

(7) $$l=\left(\begin{array}{c} l_{L}\\ \nu_{l}\\ {\bar{l}}_{R} \end{array}\right),\;l\in \left(1,\bar{3},0\right),\;l=e,\;\mu ,\;\tau .$$

As discussed in [4,7], these assignments of quarks and leptons lead to the cancellation of the anomaly of $SU{\left(3\right)}_{L}$, while the $SU{\left(3\right)}_{C}$ one is cancelled as happens in the SM, i.e., through a complete balance between left-handed colour triplets and right-handed anti-triplets in the quark sector.

The electroweak symmetry breaking of this 331 model occurs through scalar fields ρ, η, and χ, which are arranged as triplets of $SU{\left(3\right)}_{L}$:

(8) $$\rho =\left(\begin{array}{c} \rho^{++}\\ \rho^{+}\\ \rho^{0} \end{array}\right)\in \left(1,3,1\right),\;\eta =\left(\begin{array}{c} \eta^{+}\\ \eta^{0}\\ \eta^{-} \end{array}\right)\in \left(1,3,0\right),\;\chi =\left(\begin{array}{c} \chi^{0}\\ \chi^{-}\\ \chi^{--} \end{array}\right)\in \left(1,3,-1\right).$$

The breaking of $SU{\left(3\right)}_{L}\times U{\left(1\right)}_{X}\to U{\left(1\right)}_{\mathrm{em}}$ is achieved in two steps. First, the vacuum expectation value (vev) of the neutral component of ρ provides mass to novel gauge bosons $Z^{\prime }$, $Y^{++}$, and $Y^{+}$ and heavy quarks *D*, *S*, and *T*. In this first step, the original gauge group $SU{\left(3\right)}_{L}\times U{\left(1\right)}_{X}$ breaks into $SU{\left(2\right)}_{L}\times U{\left(1\right)}_{Y}$. In the second step, it is $\chi^{0}$ and $\eta^{0}$ that receive a vev, and one has the usual breaking from $SU{\left(2\right)}_{L}\times U{\left(1\right)}_{Y}$ to $U{\left(1\right)}_{\mathrm{em}}$.

In detail, the scalar potential reads as follows:

(9) $$\begin{array}{cc} V & =m_{1}\;\rho^{*}\rho +m_{2}\;\eta^{*}\eta +m_{3}\;\chi^{*}\chi \\ \; & \;+\;\lambda_{1}{\left(\rho^{*}\rho \right)}^{2}+\lambda_{2}{\left(\eta^{*}\eta \right)}^{2}+\lambda_{3}{\left(\chi^{*}\chi \right)}^{2}\\ \; & \;+\;\lambda_{12}\rho^{*}\rho \;\eta^{*}\eta +\lambda_{13}\rho^{*}\rho \;\chi^{*}\chi +\lambda_{23}\eta^{*}\eta \;\chi^{*}\chi \\ \; & \;+\;\zeta_{12}\rho^{*}\eta \;\eta^{*}\rho +\zeta_{13}\rho^{*}\chi \;\chi^{*}\rho +\zeta_{23}\eta^{*}\chi \;\chi^{*}\eta \\ \; & \;+\;\sqrt{2}f_{\rho \eta \chi }\rho \;\eta \;\chi . \end{array}$$

The neutral component of each triplet acquires a vev and can be expanded as

(10) ρ0=12vρ+12Reρ0+iImρ0

(11) η0=12vη+12Reη0+iImη0

(12) χ0=12vχ+12Reχ0+iImχ0.

As detailed in [1], one first determines the potential minimization conditions and then, after spontaneous symmetry breaking, the gauge and the mass eigenstates of ρ, η, and χ. The explicit expression of the mass matrices of the scalar sector, both neutral and charged, are provided in [1], and we do not report them here for the sake of brevity.

As anticipated in the Introduction and discussed in detail in [2], it is necessary to add to the scalar sector a $SU{\left(3\right)}_{L}$ sextet in order to provide masses to leptons. This implies that the particle spectrum of the bilepton model includes doubly charged Higgs bosons ($H^{\pm \pm }$) capable of decaying into same-sign lepton pairs. In other words, decays like $H^{\pm \pm }\to l^{\pm }l^{\pm }$ would be evidence of the presence of sextet representation of $SU{\left(3\right)}_{L}$.

Still on decays of doubly charged scalars, as pointed out in [2], in principle, as for the Standard Model Higgs, one should have amplitudes proportional to the Yukawa coupling, hence to the masses of the final-state particles. However, for the sake of generality and putting vector and scalar bileptons on the same footing, following [2], I shall consider a scenario where the branching ratios of doubly charged Higgs bosons are not proportional to the mass, but, referring, e.g., to decays into same-sign lepton pairs, one has $\mathrm{BR}\left(Y^{\pm \pm }\to l^{\pm }l^{\pm }\right)≃\mathrm{BR}\left(H^{\pm \pm }\to l^{\pm }l^{\pm }\right)$, $Y^{\pm \pm }$ being vector bileptons.

After electroweak symmetry breaking, one ends up with a rich Higgs sector. In detail, we have 5 scalar Higgs bosons, one of them being the Standard Model with mass about 125 GeV and 4 neutral pseudoscalar Higgs bosons, out of which 2 are the Goldstones of the *Z* and $Z^{\prime }$ massive vector bosons. Furthermore, one has 6 charged Higgses, 2 of which are the charged Goldstones and 3 are doubly charged Higgses, 1 of which is a Goldstone boson.

As the main goal of this investigation is the phenomenology of doubly charged vectors and scalars, we point out that Ref. [2] contains a thorough discussion of the vertices where pairs $Y^{\pm \pm }Y^{\pm \pm }$ or $H^{\pm \pm }H^{\pm \pm }$ are involved. We do not report the formulas in the present contribution for brevity and refer to [2] for such couplings.

## 3. Phenomenology at the LHC and Future Colliders

### 3.1. Leptonic Decays of Bileptons

**Figure 1:**
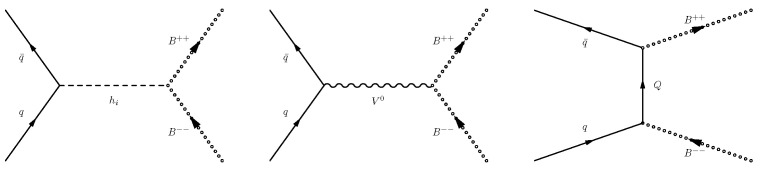
Characteristic diagrams for the production of bilepton pairs in hadron collisions.

In this section, I present the main results contained in [1,2,3] regarding the phenomenology of doubly charged scalars and vectors at the LHC (13 or 14 TeV) and future colliders, namely FCC-hh. Typical contributions to bilepton production in hadron collisions are presented in Figure 1: an initial-state $q\bar{q}$ pair annihilates and a $B^{++}B^{--}$ pair, *B* being a doubly charged vector or scalar, is produced. As can be seen, bilepton-pair production can be mediated by the exchange of, e.g., a neutral Higgs or a vector (*Z*, $Z^{\prime }$, or photon) in the *s*-channel, or a heavy TeV-scale quark, charged 5/3, in the *t*-channel. Ref. [1] also discusses the production of bilepton pairs in association with jets and presents some typical diagrams for such processes as well.

In detail, Refs. [1,2] account for decays of bileptons into same-sign lepton pairs, say $Y^{++}\to \mu^{+}\mu^{+}$, while [3] deals with non-leptonic decays, i.e., decays into a light (SM) quark (antiquark) and a heavy Tev-scale antiquark (quark), e.g., $Y^{++}\to T\bar{b},\bar{D}u$. The results are presented for a few benchmarks, determined in such a way as to be not yet excluded by the experimental searches although capable of yielding a remarkable cross section and number of events.

In order to determine the benchmarks and scan the parameter space, we had to implement the bilepton model in the SARAH 4.9.3 code [10]. In particular, Refs. [1,2] carry out a phenomenological investigation for a bilepton mass about 880 GeV, just below the experimental exclusion limit. In Ref. [2], where the phenomenologies of vector and scalar bileptons are compared, one sets the masses to the same value:

(13) $$M_{Y^{++}}≃M_{H^{++}}≃878.3\;\mathrm{GeV},$$

while exotic Higgs bosons, $Z^{\prime }$, and heavy quarks are assumed to have masses well above 1 TeV; hence, they are too heavy to contribute to any bilepton phenomenology (see [2] for their actual values in the reference points). One can then explore the process

(14) $$pp\to Y^{++}Y^{--}\left(H^{++}H^{--}\right)\to \left(l^{+}l^{+}\right)\left(l^{-}l^{-}\right),$$

setting the following cuts on final-state lepton transverse momentum ($p_{T}$), rapidity (η), and invariant opening angle:

(15) $$p_{T,l}>20\;\mathrm{GeV},\left|\eta_{l}\right|<2.5,\Delta R_{ll}>0.1.$$

In [1,2], one assumes democratic leptonic branching ratios of bileptons, namely

$\mathrm{BR}\left(Y^{++}\to l^{+}l^{+}\right)≃\mathrm{BR}\left(H^{++}\to l^{+}l^{+}\right)≃1/3$, for any lepton flavour (*e*, μ or τ).

After the cuts are applied, the leading-order cross sections of processes in Equation (14), computed by means of MadGraph 2.6.1 [11] at $\sqrt{s}=13$ TeV, read as follows:

(16) σ(pp→YY→4l)≃4.3fb;σ(pp→HH→4l)≃0.3fb.

At 14 TeV, one has instead σ(pp→YY→4l)≃6.0 fb and σ(pp→HH→4l)≃0.4 fb. As discussed in [2], the higher cross section in the case of vector-pair production can be explained in terms of the bilepton helicity. In the case of doubly charged Higgs production, only the amplitudes where the intermediate vectors (γ, *Z*, $Z^{\prime }$) have helicity zero contribute, while, in case of doubly charged vectors, all helicities 0 and ±1 play a role. For processes mediated by scalars, $Y^{++}$ and $Y^{--}$ can still rearrange their helicities in a few different ways to achieve angular-momentum conservation and a total vanishing helicity in the centre-of-mass frame. Similar results are also found in [12], where the authors investigated vector and scalar bilepton pairs at hadron colliders at parton level in the LO approximation.

**Figure 2:**
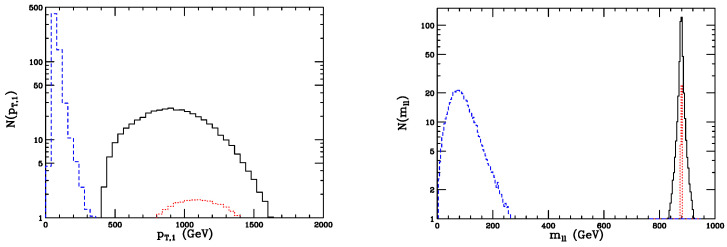
Distributions of the transverse momentum of the hardest lepton (**left**) and of the same-sign lepton invariant mass (**right)**. The solid histograms are the spectra yielded by vector bileptons, the dots correspond to scalar doubly charged Higgs bosons, and the blue dashes to the ZZ Standard Model background.

As for backgrounds, as pointed out in [1,2], the main one is due to same-sign lepton-pair production mediated by a *Z*-boson pair, i.e.,

(17) $$pp\to ZZ\to \left(l^{+}l^{-}\right)\left(l^{+}l^{-}\right),$$

while processes mediated by neutral Higgs pairs are negligible due to the tiny coupling of the Higgs with leptons. After setting the cuts, the LO cross section of the process (17) is provided by σ(pp→ZZ→4l)≃6.1 fb at 13 TeV and 6.6 fb at 14 TeV. For an integrated luminosity L=300 fb^−1^, at 13 TeV, one has N(YY)≃1302 lepton pairs mediated by doubly charged vectors, while scalars yield N(HH)≃120 and the ZZ background N(ZZ)≃1836 events. At 14 TeV and L=3000 fb^−1^, such numbers read N(YY)≃17880, N(HH)≃1260, and N(ZZ)≃19740. For *S* signal and *B* background events, one can define a significance (in units of standard deviations)

(18) $$s=\frac{S}{\sqrt{B+\sigma_{B}^{2}}},$$

where $\sigma_{B}$ is the systematic error on *B* gauged about $\sigma_{B}≃0.1B$ in [2]. Following [13], the denominator of the significance (18) sums in quadrature the intrinsic statistical fluctuation of the background $\sqrt{B}$ and the uncertainty in the background $\sigma_{B}$, obtaining $s=S/\sqrt{\sqrt{B^{2}}+\sigma_{B}^{2}}$. One can then find a significance s≃6.9 for vector pairs at 13 TeV and L=300 fb^−1^ and s=0.6 for scalars, which clearly means that only doubly charged vector bileptons may possibly be visible at 13 TeV. At 14 TeV and high integrated luminosity, one has s≃9 for $Y^{++}Y^{--}$ and s≃0.64 for $H^{++}H^{--}$ production. Reference [2] explores several distributions of relevant leptonic observables, yielded by vector and scalar bileptons, as well as ZZ background. For the sake of conciseness, we present in Figure 2 only those referring to the hardest-lepton transverse momentum $p_{T,1}$ and the same-sign lepton invariant mass.

As for the transverse momentum $p_{T,1}$, the ZZ distribution is sharp and peaked at low $p_{T}$, while those yielded by the HH and YY bileptons are much broader and peak at about 1 TeV. In fact, the background *Z* bosons are much lighter than bileptons and decay into different-sign lepton pairs, while $Y^{\pm \pm }$ and $H^{\pm \pm }$ decay into same-sign electrons and muons. Furthermore, for every value of $p_{T}$, the HH spectrum is well below the YY one.

Regarding the same-sign lepton invariant mass $m_{ll}$, as expected, the 331 signal peaks at $m_{ll}≃900$ GeV, while the *Z*-background distribution is instead a broad spectrum, significant up to about 350 GeV and maximum around 70 GeV. The signal spectra are rather narrow: the authors of [2] quoted $Y^{++}$ and $H^{++}$ widths about 7 GeV and 400 MeV, hence much smaller than their masses.

Before concluding this subsection, one can then point out that, as should have been expected due to the obtained significances, the distributions in Figure 2, as well as those published in [2], seem to show that discriminating the 331 signal from the background should be feasible, with doubly charged vectors dominating over scalars.

### 3.2. Non-Leptonic Decays of Bileptons

While possible decays into same-sign lepton pairs would be the ‘smoking gun’ for bilepton discovery at the LHC, depending on the mass spectrum, it is also possible that vector and scalar bileptons could well decay into non-leptonic final states, such as a TeV-scale heavy quark and a light quark. This was in fact the main purpose of the exploration in [3], which I shall summarize hereafter.

Unlike Refs. [1,2], the more recent work in [3] took advantage of the results of Ref. [14], where the authors, by using renormalization group arguments, provided the estimate $m_{Y}=\left(1.29\pm 0.06\right)$ TeV for the bilepton mass. Making use of this finding, Ref. [3] concentrated on doubly charged vectors $Y^{\pm \pm }$ and considered two benchmark cases: one scenario with all heavy quarks *D*, *S*, and *T* lighter than $Y^{\pm \pm }$ and another one where only the mass of *D* is lower than $m_{Y}$, while *S* and *T* are heavier. More precisely, the first benchmark, labelled BM I in [3], sets all TeV-scale quark masses to 1 TeV, i.e.,

(19) $$m_{D}=m_{S}=m_{T}=1\;\mathrm{TeV},$$

while, in the second one, i.e., BM II, one has the following mass values:

(20) $$m_{D}=1.2\;\mathrm{TeV},\;m_{S}=1.5\;\mathrm{TeV},\;m_{T}=1.5\;\mathrm{TeV}.$$

Both BM I and BM II are consistent with a light SM-like Higgs boson with mass about 125 GeV; all the other BSM particles have masses much above 1 TeV; therefore, they are not relevant for bilepton phenomenology.

Unlike Refs. [1,2], wherein bileptons could only decay leptonically, in the benchmark points of [3], one has substantial branching fractions into both leptonic and hadronic final states. In detail, one has

(21) $$\mathrm{BR}\left(Y^{++}\to l^{+}l^{+}\right)≃20.6\%\;\left(\mathrm{BM}\;I\right),\;32.5\%\left(\mathrm{BM}\;\mathrm{II}\right),$$

for each lepton flavour l=e,μ,τ, and

(22) $$\mathrm{BR}\left(Y^{++}\to u\bar{D},c\bar{S},T\bar{b}\right)≃12.7\%\;\left(\mathrm{BM}\;I\right),\;\mathrm{BR}\left(Y^{++}\to u\bar{D}\right)≃2.5\%\;\left(\mathrm{BM}\;\mathrm{II}\right).$$

The total bilepton widths instead read as follows:

(23) $$\Gamma \left(Y^{\pm \pm }\right)≃17.9\;\mathrm{GeV}\;\left(\mathrm{BM}\;I\right);\;\Gamma \left(Y^{\pm \pm }\right)≃11.4\;\mathrm{GeV}\;\left(\mathrm{BM}\;\mathrm{II}\right).$$

The larger width in BM I is clearly due to the fact that decays into final states with all three heavy quarks *D*, *S*, and *T* are permitted.

Before presenting some numerical results, one should also explore the phenomenology of TeV-scale quark decays. In BM I, the heavy quarks exhibit three-body decays into a Standard Model quark and a same-sign lepton pair or a lepton–neutrino pair, through a virtual bilepton, with the following branching fractions:

(24) $$\mathrm{BR}\left(D\left(S\right)\to u\left(c\right)l^{-}l^{-}\right)≃\mathrm{BR}\left(D\left(S\right)\to d\left(s\right)l^{-}\nu_{l}\right)≃16.7\%\;\left(\mathrm{BM}\;I\right).$$

In BM II, *S* and *T* are heavier than singly and doubly charged bileptons and can therefore decay into final states with a real $Y^{\pm }$ or $Y^{\pm \pm }$. While the *D* rates are the same as in BM I, i.e., Equation (24), *S* can decay into real bileptons as follows:

(25) $$\mathrm{BR}\left(S\to cY^{--}\right)≃50.5\%,\;\mathrm{BR}\left(S\to sY^{-}\right)≃49.5\%\;\left(\mathrm{BM}\;\mathrm{II}\right).$$

As for *T*, charged 5/3, its decay rates are

(26) $$\begin{array}{c} \mathrm{BR}\left(T\to bl^{+}l^{+}\right)≃19.4\%,\;\mathrm{BR}\left(T\to tl^{+}{\bar{\nu }}_{l}\right)≃13.9\%\;\left(\mathrm{BM}\;I\right); \end{array}$$

(27) $$\begin{array}{c} \mathrm{BR}\left(T\to bY^{++}\right)≃64.6\%,\;\mathrm{BR}\left(T\to tY^{+}\right)≃35.4\%\;\left(\mathrm{BM}\;\mathrm{II}\right). \end{array}$$

The total decay widths are provided by

(28) $$\begin{array}{c} \Gamma \left(D\right)≃\Gamma \left(S\right)≃3.4\times 10^{-3}\;\mathrm{GeV},\;\Gamma \left(T\right)≃3.0\times 10^{-3}\;\mathrm{GeV}\;\left(\mathrm{BM}\;I\right); \end{array}$$

(29) $$\begin{array}{c} \Gamma \left(D\right)≃1.3\times 10^{-2}\;\mathrm{GeV},\;\Gamma \left(S\right)≃1.5\;\mathrm{GeV},\;\Gamma \left(T\right)≃1.1\;\mathrm{GeV}\;\left(\mathrm{BM}\;\mathrm{II}\right). \end{array}$$

In other words, in BM I, all TeV-scale quarks have a pretty small width of the order $O(10^{-3}\;\mathrm{GeV})$; in BM II, *D* is still quite narrow, having a width $O(10^{-2}\;\mathrm{GeV})$, while the widths of *S* and *T* are of the order of 1 GeV since they are capable of decaying into states with real bileptons.

The production cross sections of bilepton pairs at the LHC (13 and 14 TeV) and FCC-hh are provided by

(30) $$\begin{array}{c} \sigma \left(pp\to Y^{++}Y^{--}\right)≃0.75\;\mathrm{fb}\;\left(\mathrm{LHC},13\;\mathrm{TeV}\right), \end{array}$$

(31) $$\begin{array}{c} \sigma \left(pp\to Y^{++}Y^{--}\right)≃1.12\;\mathrm{fb}\;\left(\mathrm{LHC},14\;\mathrm{TeV}\right), \end{array}$$

(32) $$\begin{array}{c} \sigma \left(pp\to Y^{++}Y^{--}\right)≃393.89\;\mathrm{fb}\;\left(\mathrm{FCC}-hh\right), \end{array}$$

with the FCC-hh cross sections about 500 and 350 times larger than the LHC ones.

Following [3], in BM I, I shall account for primary decays of $Y^{\pm \pm }$ into quarks *T*, which further decay into a bottom quark and a same-sign muon pair, hence a final state with four *b*-flavoured jets and two same-sign muon pairs:

(33) $$pp\to Y^{++}Y^{--}\to \left(T\bar{b}\right)\left(\bar{T}b\right)\to \left(b\bar{b}\mu^{+}\mu^{+}\right)\left(b\bar{b}\mu^{-}\mu^{-}\right)\;\left(\mathrm{BM}\;I\right).$$

In reference point BM II, I shall instead explore primary decays into quarks *D* and final states with four *u*-quark initiated light jets accompanied by four muons (4u4μ):

(34) $$pp\to Y^{++}Y^{--}\to \left(\bar{D}u\right)\left(D\bar{u}\right)\to \left(u\bar{u}\mu^{+}\mu^{+}\right)\left(u\bar{u}\mu^{-}\mu^{-}\right)\;\left(\mathrm{BM}\;\mathrm{II}\right).$$

In Ref. [3], a few representative diagrams of processes (33) and (34) are presented as well.

A first rough estimation of the predicted number of events at the LHC and FCC-hh can be obtained by multiplying the inclusive cross sections in Equation (30) by the relevant branching ratios, assuming a perfect tagging efficiency and no cut on final-state jets and leptons. At the LHC, one obtains

(35) $$\begin{array}{c} \sigma \left(pp\to YY\to 4b4\mu \right)≃4.55\times 10^{-4}\;\mathrm{fb}\;\left(\mathrm{LHC},13\;\mathrm{TeV},\;\mathrm{BM}\;I\right), \end{array}$$

(36) $$\begin{array}{c} \sigma \left(pp\to YY\to 4b4\mu \right)≃6.80\times 10^{-4}\;\mathrm{fb}\;\left(\mathrm{LHC},14\;\mathrm{TeV},\;\mathrm{BM}\;I\right), \end{array}$$

(37) $$\begin{array}{c} \sigma \left(pp\to YY\to 4u4\mu \right)≃1.31\times 10^{-5}\;\mathrm{fb}\;\left(\mathrm{LHC},13\;\mathrm{TeV},\;\mathrm{BM}\;\mathrm{II}\right), \end{array}$$

(38) $$\begin{array}{c} \sigma \left(pp\to YY\to 4u4\mu \right)≃2.03\times 10^{-5}\;\mathrm{fb}\;\left(\mathrm{LHC},14\;\mathrm{TeV},\;\mathrm{BM}\;\mathrm{II}\right). \end{array}$$

Such cross sections are too small to see any event at 300 fb^−1^ and at 3000 fb^−1^ (HL-LHC), even before imposing any acceptance cut. Therefore, the investigation in [3] discarded the LHC environment and the analysis was concentrated on FCC-hh, where the cross sections are remarkable:

(39) $$\begin{array}{c} \sigma (pp\to YY\to 4b4\mu )≃0.24\;\mathrm{fb}\;(\mathrm{FCC}-hh,\;\mathrm{BM}\;I), \end{array}$$

(40) $$\begin{array}{c} \sigma \left(pp\to YY\to 4u4\mu \right)≃6.87\times 10^{-3}\;\mathrm{fb}\;\left(\mathrm{FCC}-hh,\;\mathrm{BM}\;\mathrm{II}\right). \end{array}$$

The scenario BM I at FCC-hh yields a few hundreds events; BM II is less promising but still worthwhile to investigate.

As for the backgrounds, one considers, above all, four *b* quarks and two *Z* bosons decaying into muon pairs (background $b_{1}$),

(41) $$pp\to bb\bar{b}\bar{b}ZZ\to bb\bar{b}\bar{b}\mu^{+}\mu^{-}\mu^{+}\mu^{-},$$

and four top quarks with the subsequent *W*s decaying into muons and requiring, as in [2], a small missing energy due to the muon neutrinos (background $b_{2}$):

(42) $$pp\to tt\bar{t}\bar{t}\to \left(bW^{+}\right)\left(bW^{+}\right)\left(\bar{b}W^{-}\right)\left(\bar{b}W^{-}\right)\to bb\bar{b}\bar{b}\mu^{+}\mu^{+}\mu^{-}\mu^{-}\nu_{\mu }\nu_{\mu }{\bar{\nu }}_{\mu }{\bar{\nu }}_{\mu }.$$

As discussed in [3], the simulation of (42) accounted for electroweak corrections as well since, as pointed out in [15], at both LO and NLO, they can contribute up to 10% of the total cross section.

Reference [3] also considered the following backgrounds with four light jets and two *Z* bosons and with two light jets, two *b*-jets, and two *Z*s:

(43) $$\begin{array}{c} pp\to jjjjZZ\to jjjj\mu^{+}\mu^{-}\mu^{+}\mu^{-},pp\to jjb\bar{b}ZZ\to jjb\bar{b}\mu^{+}\mu^{-}\mu^{+}\mu^{-}. \end{array}$$

In Equation (43), *j* is either a light-quark or gluon-initiated jet, mistagged as a *b*-jet.

I cluster the final states of hadrons in four jets according to the $k_{T}$ algorithm and apply the following acceptance cuts on jets and muons:

(44) $$\begin{array}{c} p_{T,j}>30\;\mathrm{GeV},\;p_{T,\mu }>20\;\mathrm{GeV},\;|\eta_{j}\left|<4.5,\right|\eta_{\mu }|<2.5,\\ \Delta R_{jj}>0.4,\;\Delta R_{\mu \mu }>0.1,\;\Delta R_{j\mu }>0.4. \end{array}$$

The cuts in (44) correspond to a conservative choice of the so-called ‘overlap removal’ algorithm used at the LHC to discriminate lepton and jet tracks at the LHC [16]. As for the four-top background (42), Ref. [3] sets the additional cut MET<200 GeV on the missing transverse energy due to the neutrinos in the final state. In [3], the MET cut was consistently set even on neutrinos coming from hadron decays.

In principle, one should account for the *b*-tagging efficiency, as well as the probability of mistagging a light jet as a *b*-jet. Such efficiencies depend on the jet rapidity, transverse momentum, and flavour; however, for an explorative analysis, like the one in [3], one can implement such effects in a flat manner, i.e., independently of the jet kinematics and of the flavour of the light jets. The *b*-tagging efficiency ($\epsilon_{b}$) and the mistag rate ($\epsilon_{j}$, with j=u,d,s,c) are then set to the following values, as in [17]:

(45) $$\epsilon_{b}=0.82\;,\;\epsilon_{j}=0.05.$$

**Figure 3:**
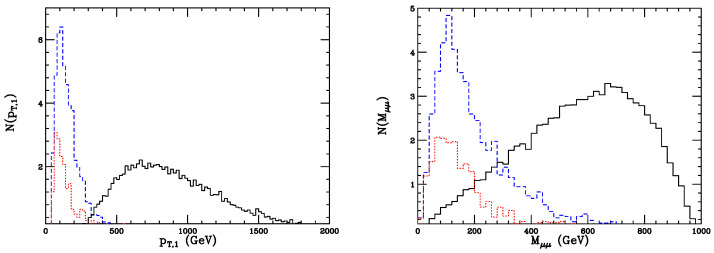
Distributions of the transverse momentum of the hardest muons (**left**) and of the same-sign muon invariant mass (**right**). The solid histograms are the signals, the dashes correspond to four tops, and the dots to the bbZZ background.

After setting such cuts, the signal (*s*) cross section of process (33) amounts to $\sigma {\left(4b4\mu \right)}_{s}≃6.24\times 10^{-2}$ fb, leading to $N{\left(4b4\mu \right)}_{s}≃90$ events at FCC-hh for an integrated luminosity L=3000 fb^−1^ and after setting all cuts and *b*-tagging efficiency. As for the backgrounds (41)–(43), one obtains $\sigma {\left(4b4\mu \right)}_{b_{1}}≃1.28\times 10^{-2}$ fb, $\sigma {\left(4b4\mu +\mathrm{MET}\right)}_{b_{2}}≃3.34\times 10^{-2}$ fb, $\sigma {\left(4j4\mu \right)}_{b_{3}}≃4.43$ fb, $\sigma {\left(2b2j4\mu \right)}_{b_{4}}≃1.34$ fb. Including also *b*-tagging and mistag efficiencies and rounding to the nearest ten, one computes the following number of background events at FCC-hh: $N{\left(4b4\mu \right)}_{b_{1}}≃20$, $N{\left(4b4\mu +\mathrm{MET}\right)}_{b_{2}}≃50$. The backgrounds $b_{3}$ and $b_{4}$ yield too few events to be significant. Regarding BM II and the decay chain (34), the cross section is about $\sigma {\left(4j4\mu \right)}_{s}≃1.88\times 10^{-3}$ fb at FCC-hh. As a result, considering that some extra suppression is due to the efficiency of jet/lepton tagging, the BM II reference point was eventually discarded in [3]. Several observables were presented in [3] for the purpose of the benchmark BM I; as for leptonic decays, in Figure 3, the hardest muon transverse momentum ($p_{T,1}$) and the invariant mass of same-sign muons ($M_{\mu \mu }$) are plotted for both signal and background. The background $p_{T,1}$ spectra are substantial only at low transverse momenta, peaking about $p_{T,1}≃100$ GeV and rapidly vanishing at large $p_{T}$, while the signal ones are broad and substantial up to $p_{T,1}≃2$ TeV. Above a few hundred GeV, the signal greatly dominates over the background; this was expected since signal muons are related to the decay of a TeV-scale resonance. Regarding $M_{\mu \mu }$, unlike the backgrounds, whose spectra peak at low values and are negligible above 500 GeV, the signal yields a broad invariant-mass spectrum, shifted towards large values and exhibiting a maximum about 700 GeV.

## 4. Discussion

I reviewed recent phenomenological work carried out in Refs. [1,2,3] within the $SU{\left(3\right)}_{C}\times SU{\left(3\right)}_{L}\times U{\left(1\right)}_{X}$, i.e., 331 model, which has, above all, appropriate features to explain the number of quark and lepton families and the asymmetry between the third and first two quark families. I worked in the framework of [4] and explored the possibility to discover doubly charged bileptons, i.e., doubly charged vectors or scalars with lepton number ±2, at the LHC and at a future 100 TeV collider FCC-hh. I first considered the production of bilepton pairs and decays into same-sign leptons, and then I accounted for non-leptonic decays too. In both cases, a few benchmarks, not yet excluded by the experimental searches, were determined to maximize the cross section at the LHC. Regarding the leptonic decays, it was found that a discovery of bileptons is feasible, with a possible signal due to vector bileptons dominating over the scalars, because of helicity arguments. Decays into non-leptonic final states are more cumbersome since bileptons decay into a heavy TeV-scale quark and a light Standard Model quark: the cross section of the resulting decay chain is too small at the LHC, even in the high-luminosity phase, to provide any signal. Nevertheless, non-leptonic decays of bileptons are expected to be visible at a future FCC-hh.

Before concluding, I wish to stress that, while the work presented here deals with the primary production of bileptons, it is certainly a very interesting scenario regarding TeV-scale quarks *T*, *S*, and *D*, which are heavier than $Y^{++}$, so they can be produced in processes like $pp\to T\bar{T}$ and decay according to, e.g., $T\to Y^{++}b$. A study of heavy-quark production and decays, along the lines of [18] but specific to the 331 model, is currently in progress [19].

In summary, as the most-studied models of new physics have provided no visible signal yet, exploring alternative scenarios is compelling. The bilepton model is certainly an appealing framework from the theoretical viewpoint, and, as summarized in this contribution, it features a rich phenomenology that may be a first indication of new physics in the next LHC run as well as HL-LHC and FCC-hh. It is then hopeful and desirable that the experimental collaborations use the results presented here and join the effort to search for bileptons at present and future colliders.

## Data Availability

The results presented in this paper are openly available in Refs. [1,2,3]. The release of the computing codes used to obtain such results is under way. For the time being, the codes are available upon request from the author.
